# Birth asphyxia, determinants, and its management among neonates admitted to NICU in Harari and Dire Dawa Public Hospitals, eastern Ethiopia

**DOI:** 10.3389/fped.2022.966630

**Published:** 2023-01-16

**Authors:** Sewmehon Amsalu, Merga Dheresa, Yadeta Dessie, Bajrond Eshetu, Bikila Balis

**Affiliations:** ^1^ Department Midwifery, College of Health and Medical Sciences, Dire Dawa University, Dire Dawa, Ethiopia; ^2^ School of Public Health, College of Health and Medical Sciences, Haramaya University, Harar, Ethiopia; ^3^ School of Nursing and Midwifery, College of Health and Medical Sciences, Haramaya University, Harar, Ethiopia

**Keywords:** birth asphyxia, determinants, management, Harari, Dire Dawa, Ethiopia

## Abstract

**Background:**

Despite a declining neonatal mortality rate globally, Ethiopia has scored 29–30 deaths per 1,000 live births. Birth asphyxia is a major contributor to neonatal mortality, where 4–9 million newborns develop birth asphyxia each year. This study aimed to assess the prevalence of birth asphyxia, its determinants, and its management among neonates admitted to the NICU in Harari and Dire Dawa public hospitals.

**Methods:**

A facility-based cross-sectional study was conducted among 409 randomly selected neonates and their index mothers admitted to neonatal intensive care units of public hospitals in Harari and Dire Dawa from June 20 to August 20, 2021. Data were collected through card review and interviewer-administered questionnaires. The collected data were entered into Epi data version 3.1 and exported to SPSS version 20 for analysis. Logistic regression models were fitted to identify factors associated with birth asphyxia. Adjusted odds ratios along with 95% CIs were estimated to measure the strength of the association, and statistical significance was declared at *p*-value <0.05.

**Results:**

One-fifth of neonates [20.8% (95% CI: 16.4, 24.6%)] had birth asphyxia. Neonates born by instrumental delivery (AOR = 2.29, 95% CI: 1.10, 4.76) and neonates born to mother with PIH (AOR = 3.49, 95% CI: 1.47, 8.27), PROM (AOR = 2.23, 95% CI: 1.17, 4.26), and chorioamnionitis (AOR = 3.26, 95% CI: 1.10, 9.61) were more likely to have birth asphyxia compared to their counterpart. Ventilation with a bag and mask 50(58.8), putting on free oxygen 19(22.4), and endotracheal intubation 15(17.6) were taken as management methods.

**Conclusion:**

One out of five neonates had birth asphyxia. This urges care providers to adhere to national guidelines of obstetrics and neonatal continuum care. They also need to decrease instrumental delivery and treat PIH, PROM, and chorioamnionitis.

## Introduction

Despite a declining neonatal mortality rate globally, striking disparities exist across regions and countries (1). For instance, neonatal mortality in sub-Saharan Africa was 10 times higher than among high-income countries (2). Similarly, the neonatal mortality rate in Ethiopia increased in the last 4 years from 29 to 30 deaths per 1,000 live births (3). Birth asphyxia is the failure of neonates to initiate and sustain breathing at birth resulting from antepartum, intrapartum, and/or postpartum events (4). According to the World Health Organization (WHO), birth asphyxia is a major contributor to 900,000 neonatal deaths each year (5).

Birth asphyxia is related to the reduced availability of skilled care during pregnancy, delivery, and postpartum periods (5). Women who received midwife-led continuity of care and health information and were regulated to international standards are 16% less likely to lose their baby and 24% less likely to experience preterm birth (6). Moreover, birth asphyxia results from fetomaternal and placental events such as maternal hemorrhage, high blood pressure, cord accidents, acute abruption, uterine rupture, intrapartum infection, and long or difficult delivery (7, 8).

Birth asphyxia is preventable with strategies such as increasing ANC coverage, skilled birth attendance, and postnatal care (9). Guidelines endorsed by WHO and the American Academy of Pediatrics for neonatal resuscitation represent a standard practice that improves outcomes in asphyxiated newborns (5). Even though the guidelines for treating birth asphyxia are well established in Ethiopia, many neonates are suffering complications (19%) (10, 11). To give adequate and quick resuscitation measures for asphyxiated neonates, understanding its determinants is vital. Also, identifying birth asphyxia suitcases and determinants is vital to controlling birth asphyxia and neonatal deaths by developing contextual interventions. Therefore, this study aimed to assess the prevalence of birth asphyxia, its determinants, and its management among neonates admitted to the NICU in Harari and Dire Dawa public hospitals in Eastern Ethiopia.

## Methods

### Study setting, design, and period

A facility-based cross-sectional study was conducted in Harari and Dire Dawa public hospitals in Eastern Ethiopia from June 20 to August 20, 2021. Dire Dawa is one of the administrative cities of Ethiopia and is located at a distance of 515 km from Addis Ababa (12). Dire Dawa has a health service coverage of 95%, with two public hospitals (Dilchora and Sabiyan) equipped with neonatal intensive care units (NICUs) (13). Also, Harari is one of the 11 regions, located 526 km from Addis Ababa (14, 15). Harari has two public hospitals (Hiwot Fana and Jugol) equipped with NICUs. The study was conducted in the NICU. The unit is run by consultants, pediatric and child health residents, and nurses.

### Sample size determination

The sample size was determined using a single-population proportion formula with assumptions; the prevalence of neonatal sepsis among neonates admitted to NICU is 52.6% according to the study conducted in Jimma (16), with 95% level of confidence = 1.96 and a margin of error = 5% (*d* = 0.05):

$$n=\frac{{\left(1.96\right)}^{2}*0.526*\left(1-0.526\right)}{{\left(0.05\right)}^{2}}=\;383.12\approx 383$$

Therefore, a sample size of 421 was used for this study with a 10% nonrespondent rate.

### Sampling techniques, study populations, and eligibility criteria

**Figure 1:**
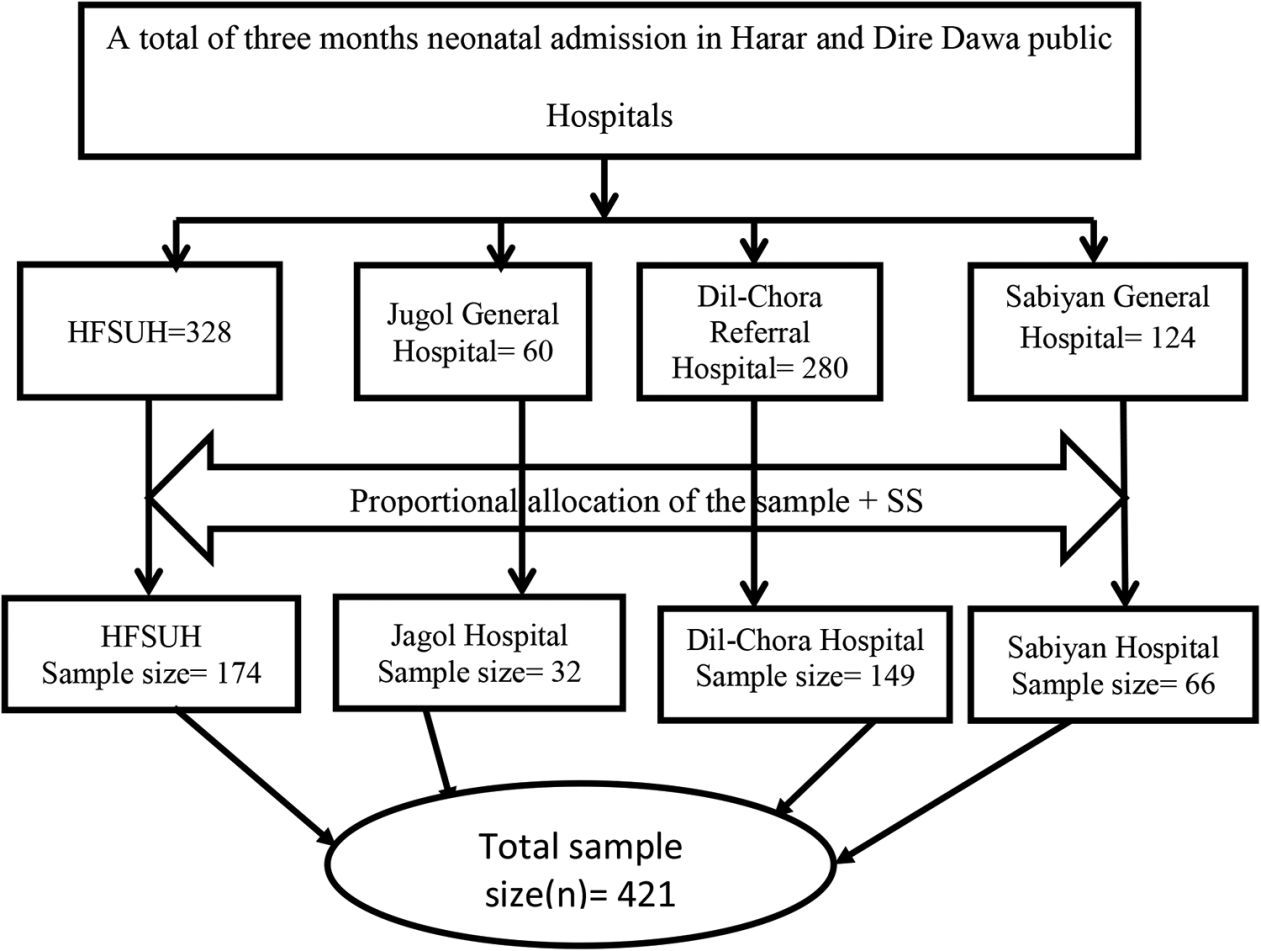
Schematic presentation of sampling procedures of mother–newborn admitted to NICUs of public hospitals in Harari and Dire Dawa, Eastern Ethiopia, 2021.

All public hospitals equipped with NICUs from Harari and Dire Dawa sites (Dilchora, Sabiyan, Hiwot Fana, and Jugol) were included conveniently. Accordingly, 968 neonates admitted to NICU in the last 3 months in both Harari and Dire Dawa public hospitals were included. Systematic sampling was used to obtain study subjects. The sampling interval was calculated by dividing the number of neonates admitted to the NICU (*N* = 968) at each hospital by the total sample size (*n* = 421). The samples from each hospital were allocated proportionally to the number of neonates admitted to the NICU at those hospitals. All participants were obtained every two intervals after the first participant (number 2) was obtained by the lottery method (Figure 1). Neonates admitted to the NICU of each hospital during the study period and mothers who gave written informed consent were our study population. Where mothers of neonates with critical conditions and who died were excluded (Figure 1).

### Data collection tools, procedures, and measurements

A structured questionnaire was adapted from different literature works (17–19). The questionnaire was designed to obtain participants' information on sociodemographic characteristics, obstetrics factors, birth asphyxia, and its management. The questionnaire was initially developed in English and translated into the local languages (Afan Oromo and Amharic) before being translated back to English. Data were collected by eight midwives using card review and interviewer-administered questionnaires. Birth asphyxia is a medical diagnosis of neonates by midwives/physicians based on the Apgar score at birth. Accordingly, an Apgar score of greater than 7 at 1 min of age was classified as no birth asphyxia, and an Apgar score of less than 7 at 1 min of age, metabolic acidosis (pH ≤ 7.0), or no breathing at 1 min of age was recorded as mild/moderate to severe birth asphyxia (20). Management modalities of birth asphyxia were extracted from the neonates' cards and records.

### Data quality control

To ensure data quality, an appropriate data collection instrument was developed. Trained data collectors were regularly supervised by principal investigators and supervisors to ensure proper data collection; all questionnaires were checked daily for completeness and consistency. A pretest was conducted on 5% of the sample size in a nearby public hospital that was not one of the main study's recruitment sites, after which the questionnaire was revised and edited, and any questions found to be unclear or ambiguous were removed or corrected accordingly.

### Data processing and analysis

The data were coded, entered into Epi data version 3.1, cleaned, and exported to SPSS version 20 for analysis. The Omnibus test and Hosmer–Lemeshow goodness-of-fit test (0.87) were used to determine the correlation between independent variables, and the multi-colinearity test was used to determine the correlation between independent variables using variable inflation factors. Descriptive statistics such as tables were used to present frequency distributions. Bivariable analysis was employed to identify factors associated with birth asphyxia. Variables with *p-*value ≤ 0.25 during bivariable analysis were entered into the multivariable logistic regression models to control for all possible confounders and to identify factors associated with birth asphyxia. The direction and strength of statistical association were measured by odds ratio with a 95% confidence interval. The odds ratio along with 95% CI were estimated to measure the strength of the association. The level of statistical significance was declared at *p*-value < 0.05.

### Ethical considerations

Ethical approval was obtained from the Institutional Review Board (IRB) of Haramaya University, College of Health and Medical Sciences (Ref. No. IHRERC/105/2021). Written permission was obtained from each of the hospital administrative bodies and unit heads. The participants were informed that their participation was entirely voluntary. Moreover, informed and written consent was obtained from each study participant (mother of a neonate).

## Results

### Sociodemographic characteristics of participants

A total of 409 neonates and their index mothers admitted to NICUs were involved in the interview; this yields a response rate of 97.1%. More than half of the mothers of neonates [225 (55.0%)] were found between the ages of 25 and 34 years. The majority of mothers of neonates [373 (91.2%)] were married, and 228 (55.7%) mothers were Oromo by ethnicity. Six out of ten mothers [247 (60.4%)] were urban residents. Regarding the educational status of mothers of neonates, 127 (31.1%) did not attend formal education and 181 (44.3%) mothers of neonates were housewives by occupation. Similarly, more than half [222 (54.3%)] were female neonates (Table 1).

**Table 1:** Socio-demographic characteristics of mothers of neonates admitted to NICUs of public Hospitals in Harari and Dire Dawa, Eastern Ethiopia (*n*=409).

Characteristics	Categories	Frequency	Percentage
Maternal age	15–24 years	153	37.4
	25–34 years	225	55.0
	35–49 years	31	7.6
Maternal religion	Muslim	285	69.7
	Orthodox	95	23.2
	Protestant	29	7.1
Maternal residence	Urban	247	60.4
	Rural	162	39.6
Maternal education	Informal	127	31.1
	Formal	282	68.9
Maternal occupation	Housewife	181	44.3
	Civil servant	72	17.6
	Merchant	11	28.8
	Student	38	9.3
Maternal ethnicity	Oromo	228	55.7
	Amhara	83	20.3
	Somali	65	15.9
	Harari	20	4.9
	Others^a^	13	3.2
Marital status	Single	13	3.2
	Married	373	91.2
	Divorced	23	5.6
Sex of the neonate	Male	187	45.7
	Female	222	54.3

^a^ Gurage, Wolayita, Silte; ETB, Ethiopian total Birr.

### Obstetrics characteristics of participants

Eight out of ten mothers [325 (79.5%)] had ANC for a recent pregnancy, and three-fifths of mothers [239 (58.4%)] were multiparous. Regarding infections of the mother, 17 (4.2%) mothers had HIV infection, 94 (23%) had UTI, and 20 (4.9%) had chorioamnionitis. More than three-sevenths of mothers [190 (46.5%)] had anemia during their recent pregnancy, and 70 (17.1%) mothers had PROM. Also, 29 (7.1%) mothers had APH and 31 (7.6%) had PIH. Eight out of ten mothers [331 (80.9%)] gave birth by SVD, and the majority of neonates [284 (69.4%)] were born at term gestational age (Table 2).

**Table 2:** Obstetrics characteristics of mothers of neonates admitted to NICUs of public Hospitals in Harari and Dire Dawa, Eastern Ethiopia, 2021 (*n* = 409).

Characteristics	Categories	Frequency	Percentage
ANC visit	Yes	325	79.5
	No	84	20.5
Parity	Primipara	170	41.6
	Multipara	239	58.4
Maternal UTI	Yes	94	23
	No	315	77
Maternal HIV status	Negative	375	91.7
	Positive	17	4.2
	Unknown	17	4.2
Anemia during pregnancy	Yes	190	46.5
	No	219	53.5
PROM	Yes	70	17.1
	No	339	82.9
APH	Yes	29	7.1
	No	380	92.9
PIH	Yes	31	7.6
	No	378	92.4
Chorioamnionitis	Yes	20	4.9
	No	389	95.1
Mode of delivery	SVD	331	80.9
	C/S	52	12.7
	Instrumental	26	6.4
Prolonged labor	Yes	390	95.4
	No	19	4.6
Gestational age at birth (weeks)	Preterm	125	30.6
	Term	284	69.4
Birth weight (kg)	Macrosomia	40	9.8
	Normal	342	83.6
	LBW	27	6.6

ANC, antenatal care; APH, antepartum hemorrhage; C/S, cesarean section; LBW, low birth weight; PIH, pregnancy-induced hypertension; PROM, premature rupture of the membrane; UTI, upper uterine infection; SVD, spontaneous vaginal delivery.

### Prevalence of birth asphyxia and its management

Of the total neonates who partook in the study, one-fifth [20.8% (95% CI: 16.4, 24.6%)] had birth asphyxia. Ventilation with a bag and mask 50(58.8), putting on free oxygen 19(22.4), and endotracheal intubation 15(17.6) were utilized as management methods (Table 3).

**Table 3:** Prevalence of birth asphyxia and its management among neonates admitted to NICU of public Hospitals in Harari and Dire Dawa, Eastern Ethiopia, 2021 (*n* = 409).

Characteristics	Categories	Frequency	Percentage
Birth asphyxia	Yes	85	20.8
	No	324	79.2
Management methods	Oxygen	19	22.4
	Ventilation with a bag and mask	50	58.8
	Endotracheal intubation	15	17.6

### Factors associated with birth asphyxia

In bivariable logistic regression, maternal age, residence, sex of the neonates, birth weight, prolonged labor, gestational age, mothers with PROM, mode of delivery, maternal anemia during pregnancy, chorioamnionitis, and PIH were factors associated with perinatal asphyxia.

However, in multivariable logistic regression, neonates born by instrumental delivery were twofold [AOR: 2.29, [95% CI: (1.10, 4.76)]] more prone to have perinatal asphyxia compared to neonates born by spontaneous vaginal delivery. The odds of perinatal asphyxia among neonates born to mothers with chorioamnionitis during pregnancy were three times [AOR = 3.26, [95% CI: (1.10, 9.61)]] higher compared to neonates whose mothers did not have chorioamnionitis during pregnancy.

Similarly, neonates who were born from a mother with PROM were two and half times [AOR = 2.23, [95% CI: (1.17, 4.26)]] more likely to develop perinatal asphyxia compared to neonates who were born from a mother without PROM. The odds of perinatal asphyxia were three times [AOR = 3.49, [95% CI: (1.47, 8.27)]] higher among neonates born from mothers with pregnancy-induced hypertension compared to their counterparts (Table 4).

**Table 4:** Determinants of birth asphyxia among neonates admitted to NICUs in Harari and Dire Dawa public hospitals, 2021.

Variables	Categories	Birth asphyxia		COR (95% CI)	AOR (95% CI)	*p*-Value
		Yes	No			
		*n* (%)	*n* (%)			
Maternal age	17–24 years	23 (27.1)	130 (40.1)	1		
	25–34 years	52 (61.2)	173 (53.4)	2.69 (1.12, 6.44)	2.12 (0.78, 5.75)	0.137
	35–49 years	10 (11.8)	21 (6.5)	1.58 (0.70, 3.57)	1.17 (0.47, 2.94)	0.727
Sex of neonate	Male	49 (57.6)	138 (42.6)	0.54 (0.33,0.88)	0.59 (0.34, 1.02)	0.063
	Female	36 (42.4)	186 (57.4)	1		
Residence	Urban	41 (48.2)	206 (63.6)	1		
	Rural	44 (51.8)	118 (36.4)	1.87 (1.15, 3.03)	1.34 (0.77, 2.33)	0.287
PROM	Yes	26 (30.6)	44 (13.6)	2.80 (1.60, 4.91)	2.23 (1.17, 4.26)	0.015
	No	59 (69.4)	280 (86.4)	1		
Prolonged labor	Yes	78 (91.8)	312 (96.3)	2.33 (0.88, 6.12)	0.51 (0.17, 1.54)	0.235
	No	7 (8.2)	12 (3.7)			
Birth weight	Normal	11 (12.9)	29 (9.0)	1		
	Low	67 (78.8)	275 (84.9)	1.55 (0.74, 3.27)	1.35 (0.58, 3.15)	0.482
	High	7 (8.2)	20 (6.2)	1.08 (0.35, 3.27)	1.57 (0.38, 6.45)	0.527
Gestational age at birth	Preterm	37 (43.5)	88 (27.2)	2.06 (1.26, 3.38)	1.69 (0.93, 3.05)	0.081
	Term	48 (56.5)	236 (72.8)	1		
PIH	Yes	13 (15.3)	18 (5.6)	3.06 (1.43, 6.55)	3.49 (1.47, 8.27)	0.004
	No	72 (84.7)	306 (94.4)	1		
Maternal anemia	Yes	36 (42.4)	154 (47.5)	0.81 (0.50, 1.31)	1.51 (0.87, 2.61)	0.139
	No	49 (57.6)	170 (52.5)	1		
Mode of delivery	C/S	18 (21.2)	34 (10.5)	0.72 (0.27, 1.89)	0.65 (0.21, 1.97)	0.449
	Instrumental	11 (12.9)	15 (4.6)	2.60 (1.37, 4.92)	2.29 (1.10, 4.76)	0.025
	SVD	56 (65.9)	275 (84.9)	1		
Chorioamnionitis	Yes	12 (14.1)	8 (2.5)	6.49 (2.56, 16.45)	3.26 (1.10, 9.61)	0.032
	No	73 (85.9)	316 (97.5)	1		

PROM, premature rupture of the membrane; SVD, spontaneous vaginal delivery; C/S, cesarean section; PIH, pregnancy-induced hypertension; COR, crude Odds Ratio; AOR, adjusted odds ratio.

## Discussion

Birth asphyxia can lead to fatal outcomes or life-long complications among neonates. Hence, information regarding the prevalence of asphyxia and its determinants is needed to develop contextual interventions that are crucial in reducing the overall newborns' morbidity and mortality. One-fifth of neonates had birth asphyxia. Similarly, instrumental delivery, PROM, PIH, and chorioamnionitis were found to be determinants of birth asphyxia.

In this study, nearly one-fifth of neonates (20.8%) admitted to NICUs in Harari and Dire Dawa had birth asphyxia. This finding was in line with the studies finding in Tigray (22.1% and 18%) (21, 22), northwest Ethiopia (19.8%) (23), and Ghana (15.1%) (24). However, it was lower than the studies found in Dilla (32.8%) (10) and Nigeria (30.1%) (25). The disparity might be related to the difference in the level of obstetrics quality care, maternal complications, and health care coverage difference. Ventilation with a bag and mask, administration of oxygen, and endotracheal intubation are the treatments taken for birth asphyxia. This implies that those health facilities are adhering to national guidelines for the treatment of birth asphyxia.

Premature rupture of the membrane (PROM) was significantly associated with birth asphyxia. This is supported by a study conducted in Uganda (26) and Ghana (24). This might be due to the low uteroplacental perfusion of the blood as a consequence of cord prolapse and abruption placenta following PROM. Also, there is a high chance of meconium aspiration syndrome, leading to airway obstruction and subsequent low APGAR score and hypoxia (21, 22).

The odds of birth asphyxia were higher among women with pregnancy-induced hypertension (PIH) compared to their counterparts. This is in agreement with the study in Nigeria (27). Also, it is consistent with reports of Gebregziabher et al. (22) and Dubie et al. (23). Neonates born from hypertensive women are at a higher risk for low uteroplacental perfusion of the blood as a result of vasospasm of large arteries that can feed 600–800 ml/min to the uterus. Moreover, anticonvulsive drugs have a depressive effect on the respiratory system of the mothers, which in turn leads to perinatal asphyxia (28, 29). Health providers need to follow PIH treatment national guidelines to decrease this adverse outcome.

Neonates born by instrumental delivery were twofold more likely to have birth asphyxia compared to neonates born by spontaneous vaginal delivery. This is consonant with reports of Kune et al. (30) and Dubie et al. (23). The reason behind this is that forceps and/or vacuum extractors may cause a defective central nervous system, which makes it difficult to initiate breathing and uterine rupture, resulting in decreased blood flow to the fetus (31–34). The instrumental operators can prevent this birth asphyxia by adhering to the indications and prerequisites of instrumental delivery recommended by national guidelines (34–36).

The odds of birth asphyxia were three times more among mothers encountering chorioamnionitis during their pregnancy, similar to what was reported in southwest Ethiopia (37), India (38), and Pakistan (39). Possibly, the presence of chorioamnionitis may cause aspiration of meconium-stained amniotic fluid to occur, which can block small airways, deactivate surfactants, and may also inhibit surfactant synthesis, resulting in birth asphyxia (40, 41).

### Strengths and limitations of the study

The strength of this study was that it was representative because it covered a larger geographical area. Moreover, this study tried to identify almost all determinants of birth asphyxia, which were modifiable with quality prenatal, intrapartum, and postpartum care, and by adhering to national guidelines of obstetrics and neonatal care. On the other hand, since this study was cross-sectional, it could not identify causation, and the sampling method included only neonates admitted to NICUs. This study did not elaborate on the management and clinical outcomes of these asphyxia neonates.

## Conclusion

In this study, one in every five neonates was affected by birth asphyxia. Instrumental delivery, PIH, PROM, and chorioamnionitis were identified as determinants of perinatal asphyxia. In this study, almost all determinants of birth asphyxia were modifiable with quality prenatal, intrapartum, and postpartum care and by adhering to national guidelines of obstetrics and neonatal care.

## Data Availability

The datasets used for analysis are available from the corresponding author on reasonable request.
